# Antimicrobial, Antifertility and Antiinflammatory Approach to Tetradentate Macrocyclic Complexes of Iron(II) and Manganese(II)

**DOI:** 10.1155/MBD.2002.347

**Published:** 2002

**Authors:** Ashu Chaudhary, D. P. Jaroli, R. V. Singh

**Affiliations:** 1 University of Rajasthan Department of Chemistry Jaipur 302004 India; 2 University of Rajasthan Department of Zoology Jaipur 302004 India

## Abstract

Some antifertility inhibitors of 18 to 24-membered tetraazamacrocyclic complexes of iron(II) and manganese(II) have been synthesised by the template condensation using 1,3-phenylenediamine with malonic acid, succinic acid, glutaric acid and adipic acid. The reaction proceed smoothly to completion. The complexes were characterized by elemental analyses, molecular weight determinations, infrared, electronic, magnetic moment, mössbaur and mass spectral studies. The elemental analyses are consistent with the formation of the complexes [M(N_4_L_n_)Cl_2_] (M = Fe(lI) or Mn(II)). All these complexes are stable and monomeric in nature as indicated by the molecular weight determinations. The spectral studies confirm the octahedral geometry around the central metal atom. The complexes have been screened *in vitro* against a number of fungi and bacteria to assess their growth inhibiting potential. The testicular sperm density and testicular sperm morphology, sperm motility, density of cauda epididymal spermatozoa and fertility in mating trials and biochemical parameters of reproductive organs have been examined and discussed.


### ANTIMICROBIAL, ANTIFERTILITY AND ANTIINFLAMMATORY APPROACH TO TETRADENTATE MACROCYCLIC COMPLEXES OF IRON(II) AND MANGANESE(II)

Ashu Chaudhary[1], D. P. Jaroli[2] and R.V. Singh*[1]

University of Rajasthan, Jaipur 302004, India [1]Department of Chemistry, [2]Department of Zoology
< kudiwal@datainfosys.net> Fax : +91-141-519221

### ABSTRACT

Some antifertility inhibitors of 18 to 24-membered tetraazamacrocyclic complexes of iron(II) and manganese(II) have been synthesised by the template condensation using 1,3-phenylenediamine with malonic acid, succinic acid, glutaric acid and adipic acid. The reaction proceed smoothly to completion. The complexes were characterized by elemental analyses, molecular weight determinations, infrared, electronic, magnetic moment, mössbaur and mass spectral studies. The elemental analyses are consistent with the formation of the complexes $[M(N_4L_n)Cl_2]$ (M = Fe(II) or Mn(II)). All these complexes are stable and monomeric in nature as indicated by the molecular weight determinations. The spectral studies confirm the octahedral geometry around the central metal atom. The complexes have been screened *in vitro* against a number of fungi and bacteria to assess their growth inhibiting potential. The testicular sperm density and testicular sperm morphology, sperm motility, density of cauda epididymal spermatozoa and fertility in mating trials and biochemical parameters of reproductive organs have been examined and discussed.

### INTRODUCTION

The fast moving and expanding development in the chemistry of coordination compounds as out lined by individual scientific backgrounds, individual interest and personal idiosyncrasies has been released due to their applicability[1-5] in diverse areas of current interest mainly in agriculture and medicine. Recently, it has been shown that the involvement of periodic elements with organic moieties having nitrogen and sulphur atoms[6-7] plays a crucial role in designing a potential molecule of specific use.

The field of macrocyclic chemistry of metals is developing very rapidly because of its variety of applications[8] and importance in the areas of coordination chemistry[9]. There has been a spectacular growth in the interest in metal complexes with tetraazamacrocyclic ligands followed by extensive work on the metal controlled template synthesis of macrocyclic species[10-12]. The development in the field of bioinorganic chemistry has also been the other important factor in spurring the growth interest in complexes of macrocyclic compounds[13]. Macrocyclc ligand systems often exhibit unusual properties and some times mimic related natural macrocyclic compounds. The complexes of polydentate macrocyclic ligands are at the fore front of bioinorganic chemistry due to their variety of geometrical forms available and the possible encapsulation of the metal ion[14,15].

The preparation and study of inorganic compounds containing biologically important ligands are made easier because certain metal ions are active in many biological processes. The fact that copper, together with magnesium, calcium, iron, zinc, chromium, vanadium and manganese are essential metallic elements and exhibit sufficient biological activity when associated with certain metal-protein complexes, participating in oxygen transport, electronic transfer reactions or the storage of ions[16], has created enormous interest in the study of systems containing these metals[17]. The inspiration acquired by the aforementioned discussion, the present paper is initiated with the preparation, characterization, and biocheical screening of some of the tetraazamacrocyclic complexes of iron(II) and manganese(II) using 1,3-phenylenediamine with different dicarboxylic acids (malonic, succinic, glutaric and adipic).

### EXPERIMENTAL

The chemicals including malonic acid, succinic acid, glutaric acid, adipic acid and 1,3-phenylenediamine were used as obtained from E. Merck. $FeCl_2.4H_2O$ and $MnCl_2.4H_2O$ (BDH) were used without further purification.

### Synthesis of the Complexes

The reaction is carried out in 1:2:2 molar ratios. An ice cold solution of metal chloride (0.99 g) in methanol (50 mL) was reacted with 1,3-phenylenediamine (corresponding to metal chloride) at O°C and put in magnetically stirred 100 mL round-bottom flask. This is followed by the addition of methanolic solution of malonic, succinic, glutaric and adipic acid (corresponding to metal chloride). The reaction mixture was stirred continuously for 10 h. The resulting solid product was recovered by filtration, washed with methanol and dried *in vacuo.* The reaction proceeded as follows:

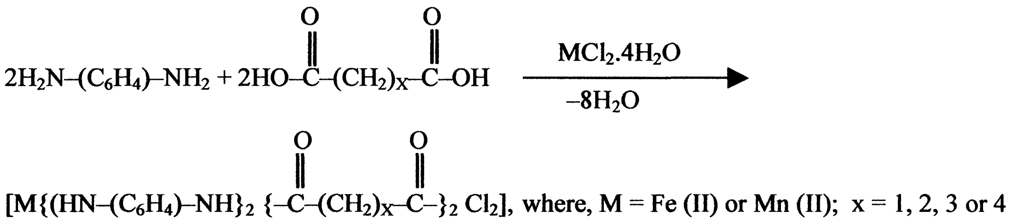

### Analytical Methods and Physical Measurements

The molecular weights were determined by the Rast Camphor Method. Conductivity measurements were made with a systronic model 305 conductivity bridge in dry dimethylformamide. The IR spectra of the solid samples were recorded as KBr discs on a Nicolet magna FTIR 550 spectrophotometer. Electronic spectra were recorded on a UV-160 A, shimadzer spectrophotometer in the range 200-600 nm using methanol as the solvent. The mass spectra of the complexes were recorded on a JEOL FX 102/DA-6000 mass spectrometer/data system using argon/xenon (6 KV, 10 mA) as the FAB gas. The accelerating voltage was 10 KV and the spectra recorded at room temperature. m-Nitrobenzyl alcohol was used as the matrix. Nitrogen and chlorine were estimated by Kjeldahl's and Volhard's method, respectively. Iron and Manganese were estimated gravimetrically.

All these complexes were recrystallized from benzene. The purity of the compounds was checked by T.L.C. on silica gel-G using anhydrous methanol and THF (1:3) as a solvent. Each of the compound moves as a single spot indicating the presence of only one component and hence their purity.

### RESULTS AND DISCUSSION

The physical properties and analytical data of the complexes are given in Table I.

**Table I : Physical Properties and Analytical Data of Tetraazamacrocyclic Complexes**

| Compound | Metal Chloride | 1,3-Phenylenediamine | Acid | Empirical formula | Mp (°C) and Colour | N Found (Calcd.) | Cl Found (Calcd.) | Metal Found (Calcd.) | Mol. Wt. Found (Calcd.) |
|---|---|---|---|---|---|---|---|---|---|
| $[Fe(N_4L_1)Cl_2]$ | $FeCl_2.4H_2O$ (0.76 g) | (0.82 g) | Malonic (0.79 g) | $C_{18}H_{16}O_4N_4Cl_2Fe$ | 192 Blackish brown | 10.50 (11.70) | 14.13 (14.80) | 11.67 (11.66) | 452 (479) |
| $Fe(N_4L_2)Cl_2]$ | $FeCl_2.4H_2O$ (0.87 g) | (0.95 g) | Succinic (1.03g) | $C_{20}H_{20}O_4N_4Cl_2Fe$ | 144 Blackish brown | 10.09 (11.06) | 13.40 (13.99) | 10.61 (11.15) | 479 (507) |
| $[Fe(N_4L_3)Cl_2]$ | $FeCl_2.4H_2O$ (0.85g) | (0.92 g) | Glutaric (1.42g) | $C_{22}H_{24}O_4N_4Cl_2Fe$ | 174 Blackish brown | 9.17 (10.47) | 12.62 (13.25) | 9.93 (10.43) | 507 (535) |
| $[Fe(N_4L_4)Cl_2]$ | $FeCl_2.4H_2O$ (0.95 g) | (1.03 g) | Adipic (1.13g) | $C_{24}H_{28}O_4N_4Cl_2Fe$ | 132 Blackish brown | 9.96 (9.95) | 11.99 (12.59) | 9.93 (9.92) | 529 (563) |
| $[Mn(N_4L_5)Cl_2$ | $MnCl_2.4H_2O$ (0.98 g) | (1.02 g) | Malonic (1.07g) | $C_{18}H_{16}O_4N_4Cl_2Mn$ | 174 Brown | 10.74 (11.72) | 14.22 (14.83) | 11.01 (11.50) | 461 (478) |
| $[Mn(N_4L_6)Cl_2$ | $MnCl_2.4H_2O$ (0.95 g) | (1.14 g) | Succinic (1.04g) | $C_{20}H_{20}O_4N_4Cl_2Mn$ | 161 Brown | 10.32 (11.07) | 13.41 (14.01) | 10.12 (10.86) | 488 (506) |
| $[Mn(N_4L_7)Cl_2$ | $MnCl_2.4H_2O$ (0.90 g) | (1.20 g) | Glutaric (0.98g) | $C_{22}H_{24}O_4N_4Cl_2Mn$ | 175 Brown | 9.19 (10.49) | 12.69 (13.28) | 10.16 (10.29) | 503 (534) |
| $[Mn(N_4L_8)Cl_2$ | $MnCl_2.4H_2O$ (1.01 g) | (1.49 g) | Adipic (1.10g) | $C_{24}H_{28}O_4N_4Cl_2Mn$ | 159 Brown | 9.12 (9.97) | 11.99 (12.02) | 9.24 (9.78) | 542 (562) |

### IR Spectra

The Infrared spectra of the starting materials and their metal complexes have been recorded. Their comparative study confirm the formation of proposed macrocyclic framework. Disappearance of the absorption bands due to hydroxyl of dicarboxylic acid and primary amino groups of 1,3-phenylenediamine, provide evidence for this cyclization. The spectra of all the complexes show a signle band in the region 3221-3277 $cm^{-1}$ which is associated with the N–H stretching mode of amide group[18]. The amide I, amide II, amide III and amide IV groups present in plane deformations are indicated by the bands at 1590-1718, 1412-1530, 1215-1271 and 632-674 $cm^{-1}$, respectively[19]. In marked, contrast to the complexes characteristic bands due to $\nu$ (M-N) vibrations exhibit at 405-468 $cm^{-1}$ unequivocally supports that four nitrogen atoms of the complexes are coordinating to the metal[20]. The infrared spectral data of the complexes are given in Table II.

**Table II : IR Spectral Data of the Tetraazamacrocyclic Complexes**

| Compound | | Amide Bands | | | | C–H | | ν(M–N) | ν(M–Cl) |
|---|---|---|---|---|---|---|---|---|---|
| | ν(N–H) | I | II | III | IV | Stretching | Bending | | |
| $[Fe(N_4L_1)Cl_2]$ | 3221 | 1605 | 1445 | 1215 | 644 | 2965 | 1434 | 440 | 356 |
| $[Fe (N_4L_2)Cl_2]$ | 3229 | 1590 | 1412 | 1266 | 625 | 2944 | 1413 | 453 | 322 |
| $[Fe (N_4L_3)Cl_2]$ | 3237 | 1597 | 1450 | 1271 | 635 | 2937 | 1428 | 475 | 334 |
| $[Fe (N_4L_4)Cl_2]$ | 3259 | 1628 | 1468 | 1243 | 630 | 2920 | 1419 | 461 | 310 |
| $[M(N_4L_5)Cl_2$ | 3230 | 1715 | 1502 | 1248 | 646 | 2895 | 1437 | 422 | 275 |
| $[Mn(N_4L_6)Cl_2$ | 3236 | 1718 | 1526 | 1230 | 667 | 2910 | 1424 | 405 | 287 |
| $[Mn(N_4L_7)Cl_2$ | 3248 | 1670 | 1511 | 1256 | 632 | 2944 | 1432 | 459 | 250 |
| $[Mn (N_4L_8)Cl_2$ | 3277 | 1685 | 1530 | 1235 | 674 | 2915 | 1438 | 468 | 293 |

**Electronic Spectra**

The electronic spectra of iron(II) complexes $[Fe(N_4L_1)Cl_2]$-$[Fe(N_4L_4)Cl_2]$ exhibit a weak intensity band in the region 856-887 nm which may be assigned to $^5T_{2g} \rightarrow {}^5E_g$ transition consistant with an octahedral geometry[21] around the iron atom.

The electronic spectra of the complexes $[Mn(N_4L_5)Cl_2]$-$[Mn(N_4L_8)Cl_2]$ display weak absorption bands in the regions 582-595, 426-451 and 377-383 nm for $^6A_{1g} \rightarrow {}^4T_{1g}$, $^6A_{1g} \rightarrow {}^4T_{2g}$ and $^6A_{1g} \rightarrow {}^4t_{1g}$, respectively. These are in fair agreement with octahedral geometry for the Mn(II) complexes[22].

**Magnetic Moment**

The observed magnetic moments for the compounds $[Mn(N_4L_5)Cl_2]$-$[Mn(N_4L_8)Cl_2]$ is 5.71-5.92 B.M. suggesting the high-spin $d^5$ configuration of octahedral geometry[23].

**$^{57}$Fe Mössbaur Spectra**

in $^{57}$Fe Mössbaur spectra the value of isomer shift (0.23-0.28 mm $S^{-1}$) and quadrupole splittings (0.65 mm $S^{-1}$) at the room temperature are characteristic of six coordinated low spin iron(II) complexes[24].

**Mass Spectra**

The mass spectrum of the compound $[Mn(C_{22}H_{24}O_4N_4)Cl_2]$ shows the molecular ion peak at m/z 534 $[M]^+$. The two coordinated chlorides are removed with a mass loss of 70. The other molecular cations during fragmentation process obtained are: 458 $[Mn(C_{16}H_{20}O_4N_4)Cl_2]^+$; 428 $[Mn(C_{16}H_{18}N_2)Cl_2]^+$ and 492 $[Mn(C_{19}H_{18}O_4N_4)Cl_2]^+$ from the loss of $C_6H_4$, $C_6H_6N_2$ and $C_3H_6$, respectively.

According to the spectral evidences the following structure can be proposed for the resulting tetraazamacrocyclic complexes in which the metal atom is in the hexa-coordinated environment.

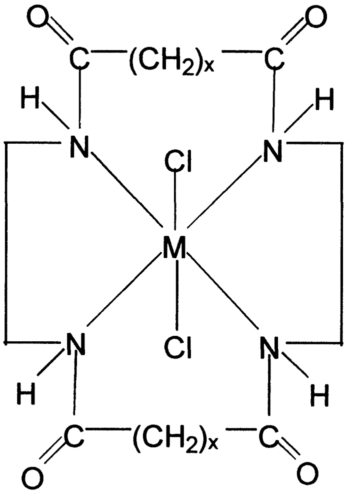

where, M = Fe (II) or Mn (II) and x = 1,2,3 or 4

### ANTIMICROBIAL ACTIVITY

Biochemical applications have greater demand nowadays. Activities of fungi and bacteria on several compounds give more important informations about complexes. So it is promoted us to screen all the macrocyctic complexes to find out which part of the molecule is actually responsible for its physiological activity.

**Antifungal Activity**

The antifungal activity has been evaluated against several fungi by the Radial Growth Method[25]. The compounds were directly mixed with the medium in 25, 50, 100 and 200 ppm concentrations. Controls were also run and three replicates were used in each case. The linear growth of the fungus was obtained by measuring the diameter of the fungal colony after four days. The amount of growth inhibition in each of the replicates was calculated by the equation (C-T) _ 100/C, where, C is the diameter of the colony on the control plate and T is the diameter of the fungal colony on the test plate.

**Antibacterial Activity**

The antibacterial activity was determined by the inhibition zone technique[26]. All the compounds were dissolved in methanol and paper discs of Whatman No. 1 paper with a diameter of 5 mm were soaked in these solutions. These discs were placed on the appropriate medium previously seeded with organisms in petri discs and stored in an incubator at 30±1°C. the inhibition zone thus formed around each disc was measured in mm after 24 hours. Data of these activities are summarised in Tables III and IV.

**Table III : Antifungal Screening Data of the Tetradentate Macrocyclic Complexes**

| Compound | Average % inhibition after 96 hours (conc. in ppm) | | | | | | | |
|---|---|---|---|---|---|---|---|---|
| | *Alternaria alternata* | | | | *Alternaria brassicae* | | | |
| | 25 | 50 | 100 | 200 | 25 | 50 | 100 | 200 |
| $[Fe(N_4L_1)Cl_2]$ | 40 | 51 | 60 | 69 | 37 | 49 | 56 | 65 |
| $[Fe (N_4L_2)Cl_2]$ | 47 | 59 | 72 | 100 | 44 | 55 | 69 | 92 |
| $[Fe (N_4L_3)Cl_2]$ | 48 | 60 | 75 | 100 | 45 | 56 | - | 100 |
| $[Fe (N_4L_4)Cl_2]$ | 53 | 65 | 80 | 100 | - | - | - | - |
| $[M(N_4L_5)Cl_2$ | 56 | 70 | 84 | 100 | 52 | 66 | 80 | 84 |
| $[Mn(N_4L_6)Cl_2$ | 53.5 | 69 | 80 | 100 | 50 | 68 | 83 | 95 |
| $[Mn(N_4L_7)Cl_2$ | 46.4 | 54 | 63 | 73 | 43 | 50 | 61 | 72 |
| $[Mn (N_4L_8)Cl_2$ | 43 | 52 | 65 | 72 | 41 | 50 | 57 | 70 |
| Standard Bavistin | 84 | 87 | 100 | 100 | 82 | 91 | 100 | 100 |

**Table IV : Bacterial Screening Data of the Tetradentate Macrocyclic Complexes**

| Compound | Diameter of inhibition zone (mm) after 24 hours (conc. in ppm) | | | | | |
|---|---|---|---|---|---|---|
| | *Xanthomonas campestris* | | | *Pseudomonas syringae* | | |
| | 500 | 1000 | 2000 | 500 | 1000 | 2000 |
| $[Fe(N_4L_1)Cl_2]$ | 7 | 11 | 15 | 12 | 14 | 19 |
| $[Fe (N_4L_2)Cl_2]$ | 5 | 8 | 10 | 9 | 11 | 14 |
| $[Fe (N_4L_3)Cl_2]$ | 6 | 8 | 12 | 7 | 7 | 11 |
| $[Fe (N_4L_4)Cl_2]$ | 4 | 6 | 10 | 10 | 12 | 15 |
| $[M(N_4L_5)Cl_2$ | - | - | - | 8 | 12 | 14 |
| $[Mn(N_4L_6)Cl_2$ | 15 | 17 | 20 | 13 | 18 | 22 |
| $[Mn(N_4L_7)Cl_2$ | 11 | 13 | 17 | 11 | 14 | 16 |
| $[Mn (N_4L_8)Cl_2$ | 10 | 11 | 13 | 12 | 17 | 20 |
| Standard Streptomycin | 3 | 5 | 6 | 2 | 3 | 5.5 |

**Mode of Action**

The results reveal that the activity increases on complexation. The newly synthesized complexes have indeed been found to be more active in inhibiting the growth of fungi and bacteria than the precursors themselves. The degradative enzymes produced by microorganism are important in host infection food deterioration and break down of organic matter[27]. The metal chelates inhibit the growth of microrganism. It is assumed that production of the enzymes is being affected as the microorganisms is unable to utilize food for itself or intake of nutrient decreases of consequently growth ceases. The greater toxicity of metal complexes than the starting material can also be explained on the basis of the chelation theory[28,29]. Chelation reduces the polarity of metal ion mainly because of partial sharing of its positive charge with the donor group and possible electron-delocalisation over the whole chelation ring. This increases the lipophilic character of the metal complex, which subsequently favours its permeation through the lipid layers of the organism cell membrane and the normal cell process being impaired.

**ANTIFERTILITY ACTIVITY**

Male rats obtained from ICMR, New Delhi were used. Animals were housed in steel cages and maintained under standard conditions (12h light/12h dark cycle, 25±3°C, 35-60% humidity) water and food were given *ad libitum.* Proven fertile male rats were taken and divided into five groups of six each. The group served as vehicle (olive oil) treated control for groups A and C starting materials (50 mg/kg b. wt.)

suspended in olive oil was given for a period of 60 days. The animals of groups B and D received same dose of $[Mn(N_4L_8)Cl_2]$ respectively for the same period. On the day sixty first, these animals were autopsied and testes, epididymis, seminal vesicle and ventral prostate were removed, fat and connective tissue cleared off and kept at –20°C until assayed for total protein, sialic acid, cholestrol, fructose and glycogen by standard laboratory techniques.

**Fertility Test**

The mating exposure tests of all the animals were performed from day $55^{th}$ to $60^{th}$. They were cohabitated in the ratio 1:3. The vaginal plug and the presence of sperm in the vaginal smear were checked for positive mating. The mated females were separated to note the implantation sites on day $16^{th}$ of pregnancy through lepratomy.

**Body and Organ Weight**

No significant change was observed in the body weight after treatment with the compounds. A significant reduction in the weight of tests, epididymis, seminal vesicle and ventral prostate was observed after treatment with both the starting materials and the compounds (Table V).

**Table V : Alternation in the Body Weight and Weight of Reproductive Organs after Treatment with Different Compounds**

| Group | Treatment | Body weight (g) | | Testes mg/100gm b.wt. | Epididymis mg/100 b.wt. | Seminal vesicle mg/ 100gm b.wt. | Ventral Prostrate mg/ 100gm b.wt. |
|---|---|---|---|---|---|---|---|
| | | Initial | Final | | | | |
| A | $FeCl_2.4H_2O$ | 192±18 | $220\pm12^c$ | $910\pm70^b$ | $320\pm20^b$ | $275\pm14^b$ | $200\pm18^a$ |
| B | $[Fe(N_4L_3)Cl_2]$ | 179±15 | $199\pm18^c$ | $650\pm70^a$ | $208\pm15^a$ | $200\pm22^a$ | $143\pm25^b$ |
| C | $MnCl_2.4H_2O$ | 175±12 | $200\pm10^c$ | $900\pm80^a$ | $305\pm22^b$ | $280\pm12^b$ | $210\pm19^a$ |
| D | $[Mn(N_4L_8)Cl_2]$ | 190±15 | $215\pm10^c$ | $700\pm70^a$ | $200\pm19^b$ | $175\pm18^a$ | $130\pm10^b$ |
| E | Control | 180±15 | $201\pm18^c$ | 1210±90 | 480±30 | 360±20 | $255\pm15^a$ |

a = P ≤ 0.05; b = P ≤ 0.001; c = P = NS

**Sperm Dynamics**

Sperm motility in cauda epididymis and sperm density in testes and cauda epididymis were significantly reduced after treatment with both the starting materials and their complexes (Table VI).

**Table VI : Altered Sperm Dynamics and Fertility Test after Treatment with Different Compounds**

| Group | Treatment | Sperm motility (%) cauda epididymis | Sperm Density (Million/ml) | | Fertility Test (%) |
|---|---|---|---|---|---|
| | | | Testes | Cauda Epididymis | |
| A | $FeCl_2.4H_2O$ | $62\pm2.8^a$ | $2.7\pm0.6^a$ | $50\pm5^b$ | 75% negative |
| B | $[Fe(N_4L_3)Cl_2]$ | $49\pm2.2^a$ | $0.9\pm0.2^b$ | $18\pm3^b$ | 88% negative |
| C | $MnCl_2.4H_2O$ | $60\pm3.5^b$ | $2.0\pm0.5^b$ | $39\pm2^b$ | 80% negative |
| D | $[Mn(N_4L_8)Cl_2]$ | $40\pm3.2^a$ | $0.8\pm0.17^a$ | $19\pm0.9^b$ | 95% negative |
| E | Control | 74±3.0 | 3.5±0.21 | 55±4 | 100% positive |

**BIOCHEMICAL PARAMETERS LEADING TO ANTIFERTILITY**

**Total Protein**

Treatment with metal salts as well as their complexes resulted in a significant reduction in the total protein contents of testes, epididymis, seminal vesicle and ventral prostate (Table VII).

**Table VII : Effects of the Complexes on Total Protein Contents of Various Reproductive Organs of Male Rats**

| Group | Treatment | Total Protein (mg/gm) | | | |
|---|---|---|---|---|---|
| | | Tests | Epididymis | Seminal vesicle | Ventral prostate |
| A | $FeCl_2.4H_2O$ | $155\pm4^a$ | $200\pm8^a$ | $185\pm9^a$ | $190\pm8^b$ |
| B | $[Fe(N_4L_3)Cl_2]$ | $110\pm4^b$ | $104\pm2^b$ | $111\pm6^b$ | $128\pm6^b$ |
| C | $MnCl_2.4H_2O$ | $150\pm5^a$ | $190\pm20^a$ | $180\pm4^b$ | $185\pm7^b$ |
| D | $[Mn(N_4L_8)Cl_2]$ | $100\pm12^b$ | $105\pm10^b$ | $105\pm8^b$ | $109\pm10^b$ |
| E | Control | 190±10 | 255±10 | 230±5 | 240±12 |

**Sialic Acid :** A significant reduction in sialic acid contents of testes, epididymis, seminal vesicle and ventral prostate was observed after the treatment in all experimental groups (Table VIII).

**Table VIII : Effect of the Complexes on Sialic Acid of Various Reproductive Organs of Male Rats**

| Group | Treatment | Sialic Acid (mg/gm) | | | |
|---|---|---|---|---|---|
| | | Tests | Epididymis | Seminal vesicle | Ventral prostate |
| A | $FeCl_2.4H_2O$ | 6.1±0.7 | 5.3±0.8 | 6.1±0.2[a] | 6.3±0.2[b] |
| B | $[Fe(N_4L_3)Cl_2]$ | 5.9±4[b] | 5.7±0.4[b] | 5.8±0.3[b] | 6.2±0.1[b] |
| C | $MnCl_2.4H_2O$ | 6.1±5[a] | 5.3±0.9[a] | 6.1±0.2[a] | 6.3±0.2[b] |
| D | $[Mn(N_4L_8)Cl_2]$ | 3.9±12[b] | 3.7±0.4[b] | 3.1±0.2[b] | 4.0±0.1[b] |
| E | Control | 8.5±0.9 | 7.3±0.8 | 7.9±0.6 | 8.1±0.8 |

**Cholesterol** : Cholesterol contents of testes were decreased significantly in all experimental groups (Table IX).

**Table IX : Effect of the Complexes on Tissue Cholesterol Glycogen and Fructose**

| Group | Treatment | Fructose (mg/gm) Seminal Vesicle | Cholesterol (mg/gm) Testes | Glycogen (mg/gm) Testes |
|---|---|---|---|---|
| A | $FeCl_2.4H_2O$ | 375±15[b] | 6.7±0.2[b] | 3.2±0.1[b] |
| B | $[Fe(N_4L_3)Cl_2]$ | 220±20[b] | 3.1±0.2[b] | 2.4±0.1[b] |
| C | $MnCl_2.4H_2O$ | 360±10[b] | 6.2±0.5[b] | 3.7±0.2[b] |
| D | $[Mn(N_4L_8)Cl_2]$ | 280±10[b] | 4.9±0.2[b] | 2.5±0.3[b] |
| E | Control | 450±30 | 8.2±0.6 | 5.0±0.3 |

**Fructose** : A significant decrease in the seminal vesicular fructose was noticed in all experimental groups (Table-X).

**Table X : Effect of Various Compounds on Carrageeman from in Rat**

| Compounds | No. of animals used | Dose (mg/pg) body wt. | Initial* vol 0.0h | Final vol* after 3h | Vol. of Oedema* | % inhibition |
|---|---|---|---|---|---|---|
| Control | 10 | 100 | 0.615 | 1.145 | 0.530 | - |
| $[Fe(N_4L_2)Cl_2]$ | 10 | 100 | 0.683 | 0.996 | 0.313 | 40.84 |
| $[Fe(N_4L_4)Cl_2]$ | 10 | 100 | 0.806 | 1.176 | 0.170 | 67.92 |
| $[Fe(N_4L_6)Cl_2]$ | 10 | 100 | 0.830 | 1.140 | 0.310 | 41.50 |
| $[Fe(N_4L_7)Cl_2]$ | 10 | 100 | 8.10 | 1.164 | 0.354 | 33.20 |
| $[Fe(N_4L_8)Cl_2]$ | 10 | 100 | 0.729 | 0.909 | 0.180 | 66.03 |

*Average of five readings

**Glycogen** : Testicular glycogen was depleted significantly in all experimental group (Table-XI).

**Conclusion** : Present study showed that oral administration of $FeCl_2.4H_2O$, $MnCl_2.4H_2O$ and their complexes resulted in the reduction of weights of testes epididymis, seminal vesicle and ventral prostate. The weight, size and secretary activities of sex accessories are closely regulated by androgen levels[30]. Reduction in sperm density and motility in cauda epididymis is of importance with regards to fertilization[31]. Significant reduction in sperm motility and sperm density was observed in treated animals. This may be due to inhibitory effect of these compounds on the enzyme oxidative phosphorylation[32]. In our observation various androgen dependent parameters that is total protein, sialic acid, fructose, cholestrol and glycogen revealed a significant decrease indicating that administration of these compounds resulted in the fall of circulating androgen[33,34]. It is concluded that the complexes of Fe(II) and Mn(II) are more effective than the metal salts in inhibiting the fertility.

**ANTIINFLAMMATORY ACTIVITY**

Antiinflammatory activity of the complexes were performed using a plethysmometer to measure carrageenan induced rat paw volume following the method of Winter et al[35]. Adult male wister albino rats (90-125 g) were fasted for 18 h but with free assess to water. Each treatment i.e. control and complexes was administered at a dose of 100 mg/kg body weight orally in 0.5% cmc suspension. Half an hour following the treatment. 0.1 ml of 1% solution of a carrageenan was injected in the right hand paw planter aponeurosis, the paw volume was measured immediately before giving carrageenan and again 3h later by means of plethysmometer. Edema was measured in a precalibrated plethysmometer as the difference between the volume of the paw measured before the 3 h after giving carrageenan. The percent inhibition of inflammation after 3h was calculated by the method of newbould[36].

The values reveal that at equal doses, the complexes $[Fe(N_4L_4)Cl_2]$ and $[Mn(N_4L_8)Cl_2]$ are more active. However the complexes $[Fe(N_4L_2)Cl_2]$, $[Mn(N_4L_6)Cl_2]$ and $[Mn(N_4L_7)Cl_2]$ possibly depressed the synthesis of the proinflammatory (vasodilator) prostaglandin, $PGE_2$ in the carrageenan pouch model of inflammation[37]. This is in consistent with the work of Lee and Lands[38] and recently confirmed by Moddox[39], who found a depression in $PGE_2$ synthesis and a concomitant increases in the antiinflammatory prostaglandin, $PGE_{2\alpha}$, following the addition of copper sulphate or chloride to seminal vesicle homogenates.

**ACKNOWLEDGEMENT**

One of the authors (AC) is thankful to CSIR, New Delhi for financial assistance in the form of SRF vide Grant No. 9/149(288)/2K2 EMR-I.

**REFERENCES**

1. R.R. Holmes. *Acc. Chem. Res.*, 22 : 190 (1989).
2. R.J.P. Williams, *Biochem. Soc. Trans.*, **18** : 989 (1990).
3. B.M. Eliot, W.N. Aldridge and J.M. Bridges, *J. Biochem.* 177 : 461 (1979).
4. H. Adams, A.N. Baileyand and D.E. Fenton. *J. Chem. Soc. Dalton Trans,* 1 : 207 (1987).
5. A.K. Saxena and F. Hüber, *Coord. Chem. Rev.*, 95 : 169 (1987).
6. M.T. Beck. *Coord. Chem. Rev.*, 3 : 91 (1968).
7. J.G.H. Dupreez, T.T.A. Gerber and O. Knoesen, *Inorg. Chim. Acta,* 132 : 241 (1987).
8. M.W. Hosseini, J.M. Lehn, S.R. Duff, K. Gu and M.P. Mertes, *J. Org. Chem.*, **52** : 1662 (1987).
9. R.M. Izan, J.S. Brandshaw, S.A. Neilsen, J.D. Lamb, J.J. Christensen and D. Sen, *Chem. Rev.,* **85** : 271 (1985).
10. M. Tadokoro, H. Sakiyama, N. Matsumoto, M. Kodera, M. Okawa and S. Kida, *J. Chem. Soc. Dalton Trans,* 313 (1992).
11. K.I. Matoda, H. Sakiyama, N. Matsumoto, H. Okawa and S. Kida, *Bull. Chem. Soc. Jpn.,* **65** : 1176 (1992).
12. A.W. Herlinger, E.M. Funk, R.F. Chorak, J.W. Siebert and E. Roco, *Polyhedron,* **13** : 69 (1994).
13. G.A. Melson, Coordination Chemsitry Of Macrocyclic Compounds. Plenum Press, New York, 1 (1979).
14. S.V. Rasokha, Y.D. Lampeak and L.M. Moloshtar, *J. Chem. Soc., Dalton Trans,* 63 (1993).
15. M. Shakir, S.P. Varkey, O.S.M. Nasmon, *Polyhedron,* **14** : 1283 (1995).
16. G. Albertin, E. Bordighon and A.A. Orio, *Inorg. Chem.*, **14** : 1411 (1975).
17. K.D. Karlin and J. Zubieta (Eds.), Copper Coordination Chemistry : Biochemical and Inorganic Perspective, Academic Press, New York, **43** (1983).
18. K. Nakamato, "Infrared and Raman Spectra Of Inorganic and Coordination Compounds", Willey, New York, **201** (1978).
19. M. Shakir and S.P. Varkey, *Polyhedron,* 14 : **1117** (1995).
20. P.S. Mane, S.G. Shirodkar, B.R. Aribad and T.K. Chondhekar, *Indian J. Chem.*, **40A** : 648 (2001).
21. A.T. Baker, P. Singh andV. Vigncvich. *Aust. J. Chem.*, **44** : 1041 (1991).
22. M.N. Patel and V.J. Patel, *Synth. React. Inorg. Met. Org. Chem.*, **19(2)** : 137 (1989).
23. M.B.H. Howlader, M.S. Islam and M.R. Karim, *Indian J. Chem.*, **39A** : 407 (2000).
24. H.A. Goodwin, *Coord. Chem. Rev.*, **18** : 314 (1976).
25. D. Singh, R.B. Goyal and R.V. Singh, *Appl. Organomet. Chem.*, **5** : 45 (1991).
26. K. Sharma, S.C. Joshi and R.V. Singh, Metal Based Drugs. **7** : 105 (2000).
27. L. Hamkin and Anagnostakis, *Mycologia,* **67** : 597 (1975).
28. A.L. Lehninger, "Biochemistry", Second Edition, 519 (1975).
29. R.S. Srivastava, *Inorg. Chim. Acta,* **56** : 165 (1981).
30. K.N. Thimmaich, W.D. Lloyd and G.T. Chandrappa, *Inorg. Chim. Acta,* **106** : 81 (1985).
31. M. Chaturvedi and V.P. Dixit, *J. Environmental Sci.*, **1** : 89 (1997).
32. J.M. Bedford, *Biol. Reprod,* **28** : 108 (1983).
33. A.K. Purohit, *Bio. Sci.*, **3** : 179 (1991).
34. S. Belwal, S.C. Joshi and R.V. Singh, *Main Group Met. Chem.*, **20** : 313 (1997).
35. P.C. Mali, *Indian J. Environmental Sci.*, **3** : 185 (1999).
36. C.A. Winter, E.A.Risely and G.W. Nuss, *Proc, Soc. Exp. Biol.* **162** : 544 (1963).
37. B.B. Newbould, *Brit J. Pharmacol.*, **21** : 157 (1963).
38. R.E. Lee and W.E.M. Land, *Biochim. Biophy Acta.*, **260** : 203 (1972).
39. I.S. Madolox, *Biochim. Biophys Acta.*, **306** : 74 (1973).

**Received: May 27, 2002 – Accepted: July 7, 2002 –**

**Accepted in publishable format: July 3, 2002**

